# Phenotypic characterisation of the cellular immune infiltrate in placentas of cattle following experimental inoculation with *Neospora caninum* in late gestation

**DOI:** 10.1186/1297-9716-44-60

**Published:** 2013-07-22

**Authors:** Germán J Cantón, Frank Katzer, Julio Benavides-Silván, Stephen W Maley, Javier Palarea-Albaladejo, Yvonne Pang, Sionagh Smith, Paul M Bartley, Mara Rocchi, Elisabeth A Innes, Francesca Chianini

**Affiliations:** 1 Moredun Research Institute, Pentlands Science Park, Bush Loan, Penicuik EH26 0PZ, UK; 2 Instituto Nacional de Tecnología Agropecuaria (INTA), EEA, Balcarce CC276, Argentina; 3 Instituto de Ganadería de Montaña (CSIC-ULE), León 24346, Spain; 4 Biomathematics & Statistics Scotland, Edinburgh EH9 3JZ, UK; 5 Royal (Dick) School of Veterinary Studies and Roslin Institute, University of Edinburgh, Edinburgh EH25 9RG, UK

## Abstract

Despite *Neospora caninum* being a major cause of bovine abortion worldwide, its pathogenesis is not completely understood. *Neospora* infection stimulates host cell-mediated immune responses, which may be responsible for the placental damage leading to abortion. The aim of the current study was to characterize the placental immune response following an experimental inoculation of pregnant cattle with *N. caninum* tachyzoites at day 210 of gestation. Cows were culled at 14, 28, 42 and 56 days post inoculation (dpi). Placentomes were examined by immunohistochemistry using antibodies against macrophages, T-cell subsets (CD4, CD8 and γδ), NK cells and B cells. Macrophages were detected mainly at 14 days post inoculation. Inflammation was generally mild and mainly characterized by CD3^+^, CD4^+^ and γδ T-cells; whereas CD8^+^ and NK cells were less numerous. The immune cell repertoire observed in this study was similar to those seen in pregnant cattle challenged with *N. caninum* at early gestation. However, cellular infiltrates were less severe than those seen during first trimester *Neospora* infections. This may explain the milder clinical outcome observed when animals are infected late in gestation.

## Introduction

The coccidian parasite *Neospora caninum* is recognized as a major cause of abortion in cattle [1,2] and has a facultative heteroxenous life cycle involving a definitive canid host (dogs, coyotes, dingoes) [3-5] and a wide range of intermediate hosts, of which cattle are the most economically important [6,7]. Infection due to neosporosis may occur postnatally following ingestion of oocysts shed in the faeces of infected canids potentially leading to exogenous transplacental transmission (horizontal transmission) [8,9] or through recrudescence of a previous infection leading to endogenous transplacental transmission of the parasite from mother to foetus via the placenta [10-12]. Regardless of transmission route, the consequences of infection may include foetal death in utero, the birth of live but clinically affected calves and the birth of clinically normal but persistently infected calves [2,13].

The pathogenesis of bovine neosporosis is complex and is not completely understood. *Neospora* is an abortifacient in cattle since the brain and heart lesions usually observed in infected foetuses may be severe enough to cause mortality [14-16] and the infection-associated placental damage can disrupt the vascular supply of nutrients leading to foetal death [17,18]. Additionally, there is evidence that *N. caninum*, like other intracellular pathogens, stimulates a cell-mediated immune response, characterized by a T helper 1 (Th1) type response [19]. However, in some instances, relatively small numbers of parasites, whilst producing mild lesions, may cause a shift from a beneficial T helper 2 (Th2) response towards a more harmful Th1 response during pregnancy, thereby inducing abortion [19,20].

Although infection with *N. caninum* is common and transplacental transmission of tachyzoites is highly efficient, only a relatively small proportion of infected cattle abort. Some of the pathological processes that transform an apparently harmless infection into a fatal disease are still unclear [16].

The clinical outcome of bovine neosporosis during pregnancy is influenced by several factors. These include the infective dose and timing of primary infection or the recrudescence of a persistent infection [18,19]. In persistently infected cows, *N. caninum* does not appear to affect the embryonic and early foetal period [21], whereas *de novo* infections in naïve pregnant cattle during early gestation are likely to be fatal to the foetus partially due to the immature foetal immune response [17,22-24]. During the second trimester of pregnancy *Neospora* infections can result in abortions or the birth of congenitally infected calves, depending in the severity of lesions [18,23,25-28]. Finally, after experimental inoculation in the final trimester of pregnancy when foetuses are more immunologically mature and able to control the infection, congenitally infected live foetuses are recovered [23,29,30].

The severity of placental damage is a determining factor in the occurrence of abortion and also important in permitting invasion of the foetus [16,18]. Improving our understanding of the host-pathogen interaction in pregnant cattle infected with *N. caninum* will help to determine the critical factors involved in disease pathogenesis and host protective immune responses. This, in turn, will help in the development of effective control strategies, especially for vaccines.

Investigating the host immune response at the materno–foetal interface may improve our understanding as to why some infected cattle abort and some do not [19]. The aim of the present study was to characterise the phenotype of the cellular immune infiltrate in the placenta of cattle experimentally inoculated with live *N. caninum* (Nc-1 strain) tachyzoites on day 210 of gestation.

## Materials and methods

### Animals and experimental design

A full description of the animals and experimental design was published previously [29]. Briefly, 15 pregnant Aberdeen Angus cross or Belgian Blue cross cattle seronegative for *N. caninum*, *Toxoplasma gondii*, Bovine Viral Diarrhoea, Infectious Bovine Rhinotracheitis Virus and *Leptospira hardjo* were oestrus synchronized and artificially inseminated as previously described [18]. Pregnancy and foetal viability were confirmed by ultrasound scanning on day 35 after insemination and again before challenge. Before the beginning of the experiment, the animals were divided into 2 groups: *N. caninum*-inoculated (*n* = 11) and negative control (*n* = 4), but were housed together until the end of the study [29]. At day 210 of gestation they were either subcutaneously inoculated with *N. caninum* tachyzoites (*N. caninum*-inoculated group) or with PBS (negative control group) over the left pre-femoral lymph node, respectively. Three *N. caninum*-inoculated and 1 negative control animal were culled at 14, 28 and 42 days post inoculation (dpi) and 2 *N. caninum*-inoculated and 1 negative control animal on 56 dpi. Dams and foetuses were subjected to *post mortem* studies [29].

This experiment was carried out with the approval of the Moredun Research Institute experiments and ethics committee and complied fully with the regulations laid down by the Home Office of Great Britain and Northern Ireland for compliance with the Animals (Scientific Procedures) Act 1986.

### Inocula

Animals from the *N. caninum*-inoculated group were subcutaneously inoculated over the left prefemoral lymph node with 2 mL of PBS containing 5 × 10^8^ live tachyzoites of the Nc-1 strain of *N. caninum* at 210 days of gestation. Tachyzoites were cultured in Vero cells and the inoculum was prepared as in previous experiments [17,18]. Four dams from the negative control group were inoculated with a similar number of Vero cells as was found in the challenge inocula in 2 mL PBS [29].

### Tissue sampling

Immediately after euthanasia, 10 randomly selected placentomes were sampled from each animal and fixed in zinc salts fixative (ZSF) (pH 7.0-7.4) for immunohistochemistry (IHC) examination, and after 3 days of fixation, tissues were processed and then embedded in paraffin-wax. Five μm-thick serial sections were trimmed from each placentome and were mounted on glass microscope slides (Superfrost® Plus, Thermo Scientific, Braunschweig, Germany) and subjected to IHC using a panel of monoclonal antibodies (mAb) as detailed in the table in Additional file 1.

### Phenotypic analysis of inflammatory cells in placental tissue

To investigate the immunopathology following inoculation with *N. caninum*, the phenotypes of the cells present in the inflammatory infiltrate were characterised. The placental tissues were sampled as previously described [31]. For each placentome sample, 5 μm-sections were cut and dewaxed in xylene and hydrated through graded ethanol solutions. Endogenous peroxidase was blocked by incubating with 3% hydrogen peroxide in methanol for 30 min at room temperature. Non-specific labelling was reduced by using 25% normal goat serum in Tris-buffered saline (TBS). Immunohistochemistry was performed using an EnVision + kit (Dako North America Inc, Carpinteria, USA). Sections were incubated overnight with mAbs (diluted in TBS) that specifically recognize cell surface molecules: EBM11 (raised against CD68 for macrophages, Dako Cytomation, Glostrup, Denmark), MMIA (CD3 for total T cells, VMRD Inc, Washington, USA), CC30 (CD4 for T helper cells, AbD Serotec, Oxford, UK), CC58 (CD8 for cytotoxic T cells, AbD Serotec), IL-A29 (γδTCR for γδ-T cells, VMRD Inc), NKp46 (CD335 for Natural killer – NK – cells, AbD Serotec), and HM57 (CD79_αcy_ for total B cells, Dako Cytomation). For details see Additional file 1. Sections of lymph nodes were used as positive control tissues.

### Scoring of the immunolabelling on the tissues

**Figure 1:**
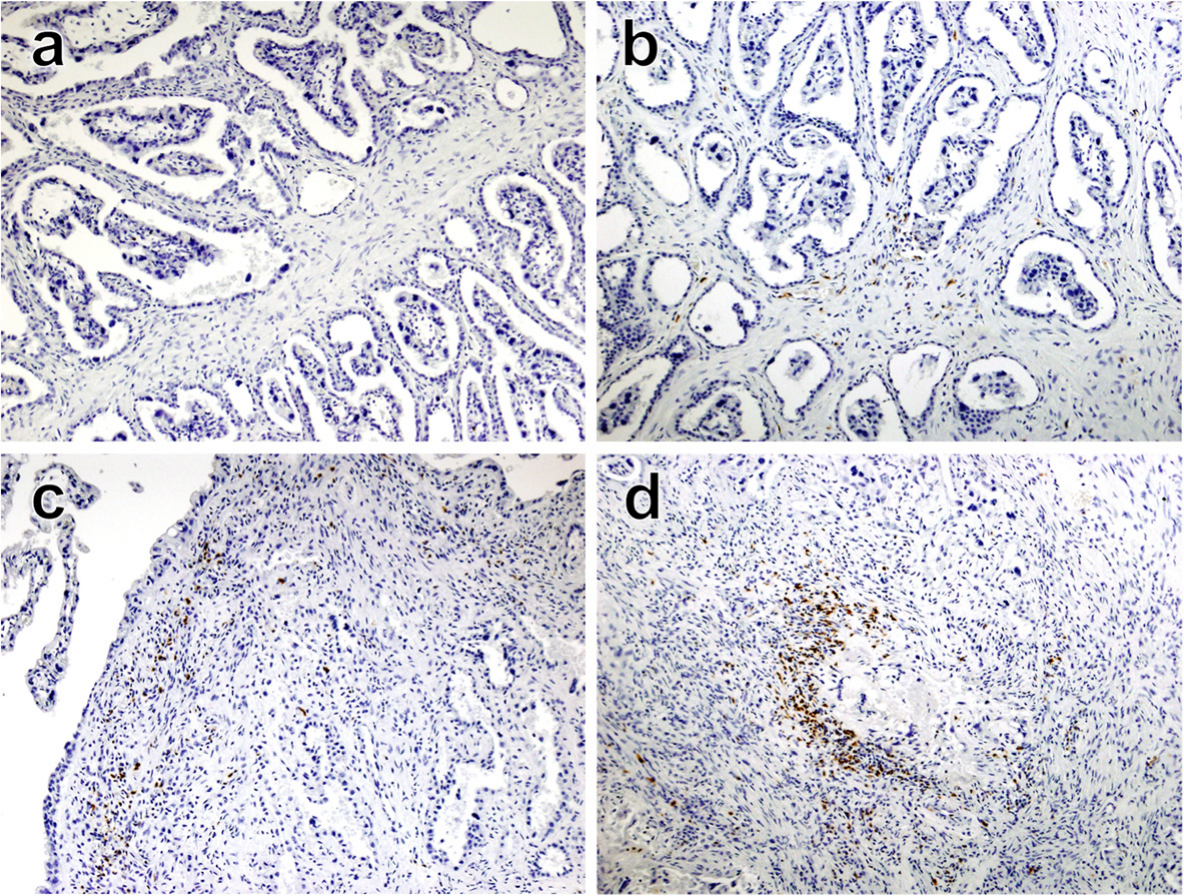
**Examples of different scores of CD3+ cell infiltration in placentomes from *N. caninum*-inoculated animals. (a)** Score 0: no infiltration of CD3^+^ cells in a placentome of a negative control cow. Counterstained with haematoxylin. Original magnification 100×. **(b)** Score 1: minimal infiltration of CD3^+^ cells in the maternal caruncles surrounding some necrotic foetal villi in the placentome of a *N. caninum*-inoculated cow culled 14 dpi. Counterstained with haematoxylin. Original magnification 100×. **(c)** Score 2: mild infiltration of CD3^+^ cells surrounding area of detachment of fetal trophoblast and mesenchyme of the maternal caruncle of the placentome of an inoculated cow culled 14 dpi. Counterstained with haematoxylin. Original magnification 100×. **(d)** Score 3: focal aggregation of CD3^+^ cells surrounding focal necrotic area in the maternal caruncle of the placentome of an inoculated cow culled 14 dpi. Counterstained with haematoxylin. Original magnification 100 × .

Similarly to the technique described by Tekin and Hansen [32], slides were blind-coded and examined for each inflammatory cell marker (listed above). To eliminate inter-operator error all slides were read by a single investigator. The whole tissue section was examined and scored for the presence and distribution of immunolabelled cells under optical microscopy conditions using various magnifications (10×, 20× and 40×). The scores were defined according to the extent of cellular infiltrate of the placentomes and whether there were associated pathological changes. In order to establish the different infiltration scores, placental tissue samples were observed and scores from 0 to 4 were established using the following criteria. Score 0: no infiltration of labelled cells or diffuse/rare infiltration of labelled cells that are not associated with pathological changes (see Figure 1a); Score 1: minimal/diffuse infiltration of labelled cells (in some cases forming small foci) associated with small necrotic areas (see Figure 1b); Score 2: mild infiltration and focal aggregation of labelled cells surrounding necrotic foci (see Figure 1c); Score 3: moderate infiltration and focal aggregation of labelled cells surrounding areas of necrosis (see Figure 1d); and Score 4: severe and large aggregation of positive cells surrounding areas of necrosis. The individual scores from 10 sampled placentomes were used to calculate a single mean score for each animal, similar to previous descriptions [33,34].

### Statistical analysis

Given the limited sample sizes and the lack of replication in the negative control group at each time point, the time factor was omitted and score values were pooled in order to gain statistical power. This was supported by results from robust Fligner and Kruskal-Wallis tests on homogeneity, respectively, variability and location parameters among *N. caninum*-inoculated animals. Then, non-parametric two-tailed Mann–Whitney tests allowing for ties were conducted on the pooled data to investigate statistically significant differences in the distribution of scores between *N. caninum*-inoculated and negative control animals for each cell type. Statistical significance was reached when *p* ≤ 0.05.

## Results

### Necropsy, histopathology, IHC and PCR

The clinical and *post mortem* examination findings from dams and foetuses were described previously by Benavides et al. [29]. Briefly, no abortions were recorded in *N. caninum*-inoculated or negative control animals. Dams inoculated with *N. caninum* showed evidence of infection and mild to minimal necrotic and inflammatory lesions were observed in the foetal and placental tissues. Furthermore, parasite antigen and DNA was detected by IHC and PCR, respectively, in foetal and placental tissues. Results from placental tissues showed differences in quantity of *N. caninum* present during the serial analysis, with no positive results at 14 dpi, a maximum number of positive placentomes at 28 dpi, and then a progressively decreasing level of positivity at 42 and 56 dpi [29]. Although the negative control dam culled at 14 dpi was later found to be *Neospora* PCR positive in different tissues, satellite markers indicated that the genotype of the parasite was different from the Nc-1 genotype used in the experimental inocula. All negative control animals did not show any significant histopathological and immunohistochemical positive finding [29].

### Phenotypic analysis of inflammatory cells in placental tissue

#### CD68^+^ cells

**Figure 2:**
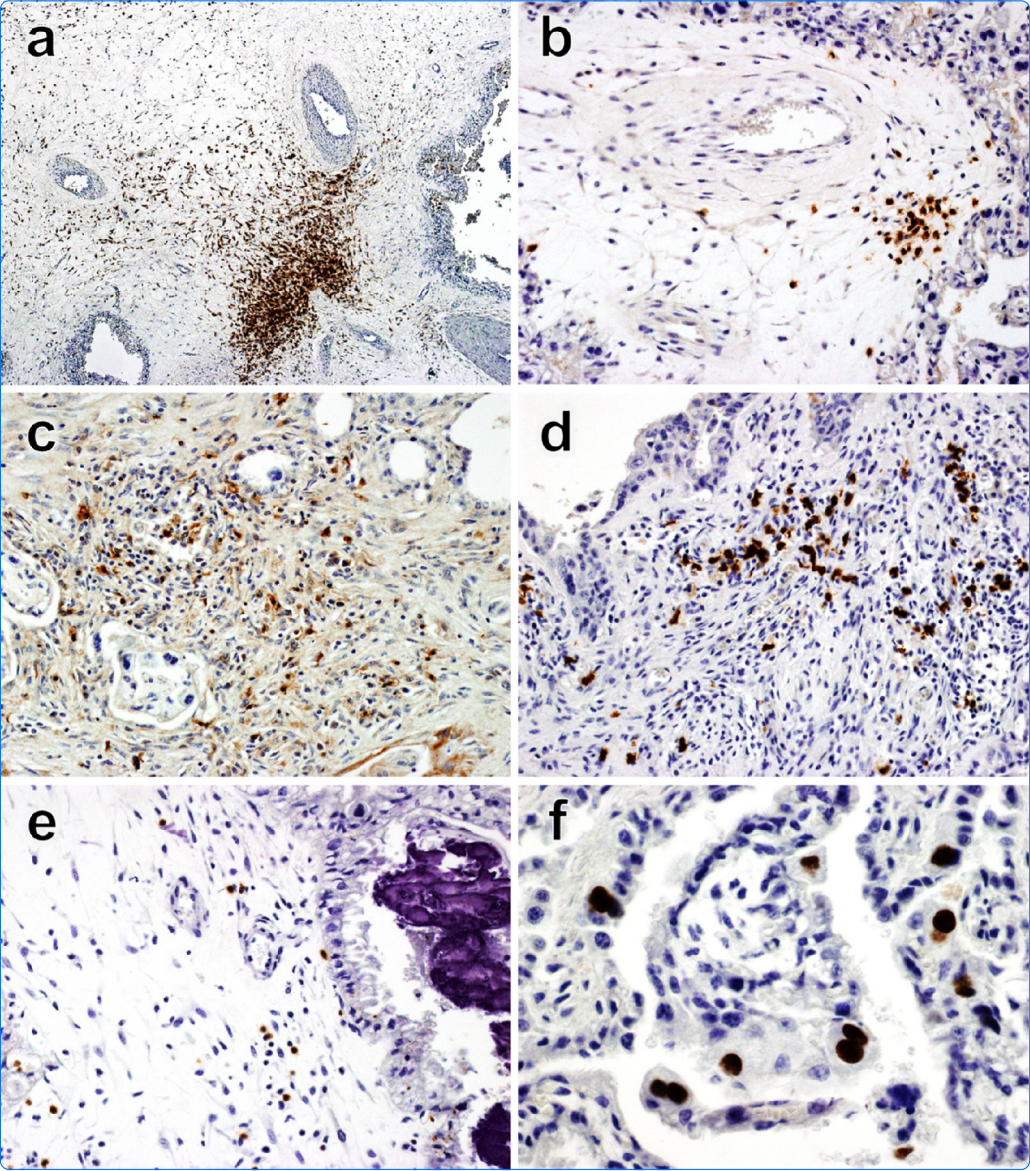
**Examples of different immune cell infiltration in placentomes from *N. caninum*-inoculated animals. (a)** Severe infiltration of macrophages in the base of maternal caruncle of a *N. caninum* inoculated cow culled 14 dpi. Counterstained with haematoxylin. Original magnification 100×. **(b)** Mild aggregate of T helper cells (CD4^+^) surrounding a blood vessel in maternal caruncle of a placentome collected from an inoculated cow culled 28 dpi. Counterstained with haematoxylin. Original magnification 200×. **(c)** Minimal infiltration of cytotoxic T-cells (CD8^+^) in a necrotic focus in maternal caruncle of a *N. caninum* challenged cow culled 28 dpi. Counterstained with haematoxylin. Original magnification 200×. **(d)** Mild aggregate of γδ T-cells in the base of maternal caruncle of an inoculated cow culled 14 dpi. Counterstained with haematoxylin. Original magnification 200×. **(e)** Rare presence of NK-cells in connective tissue in the maternal caruncle surrounding necrotic and mineralized foetal villi, observed in a placenta of an inoculated cow culled 28 dpi. Counterstained with haematoxylin. Original magnification 200×. **(f)** Rare presence of CD79_αcy_^+^ cells aligned with endometrial epithelial cells in the maternal caruncle and in some foetal villi, observed in the placentome of a *N. caninum* inoculated cow culled 28 dpi. Original magnification 400×. Counterstained with haematoxylin.

**Figure 3:**
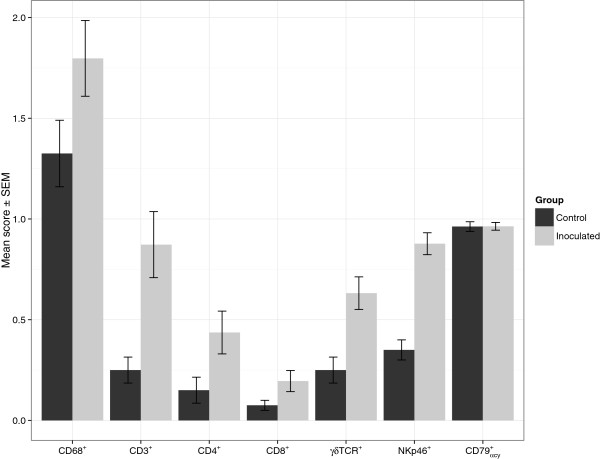
**Mean of the infiltration scores of the different phenotype of inflammatory cells on placentomes.** CD68^+^ (macrophages), CD3^+^ (total T cells), CD4^+^ (T helper), CD8^+^ (cytotoxic T cells), γδTCR^+^ (γδ T cells), NKp46^+^ (NK cells) and CD79_αcy_^+^ labelled cells in the placentas. Numbers in the horizontal axis represent days post inoculation (dpi). Error bars indicate standard error of the means (SEM).

Macrophages were observed in the placentas of all the *N. caninum*-inoculated animals throughout the period of study. The infiltrate of macrophages was more evident in the *N. caninum*-inoculated animals culled at 14 dpi. It ranged from mild to moderate and was concentrated at the base of the caruncles and between the endometrial glands. Large labelled cells in the connective tissue of the maternal caruncle (not always associated with pathological changes) (see Figure 2a) were also observed. In some cases, macrophages infiltrated necrotic foetal villi. The placentas of *N. caninum*-inoculated animals culled at 28 dpi contained a milder infiltrate of macrophages compared to those sampled at 14 dpi. This was characterised by a minimal diffuse infiltrate at the caruncle base, with some large labelled cells in the caruncle stalk connective tissue, generally not associated with any other lesions. At 42 and 56 dpi the infiltrate of macrophages was heavier than at 28 dpi, but still not as pronounced as in the placentas sampled at 14 dpi. At 42 and 56 dpi, the macrophages mainly surrounded small areas of necrosis in the maternal caruncle and in necrotic foetal villi. They also infiltrated the base of caruncles but were not associated with any lesions. Rare non-aggregated CD68^+^ cells were observed in placentas of negative control animals. The observed differences in the scores of macrophage infiltration between *N. caninum*-inoculated and negative control groups (Figure 3) were not statistically significant (*p* = 0.0987).

#### CD3^+^ cells

In 10 to 70% of the sampled placentomes of the *N. caninum*-inoculated dams culled at 14 dpi there was a minimal to mild infiltrate of CD3^+^ cells in areas of necrosis in the maternal caruncle and sporadically surrounding necrotic foetal villi, occasionally forming little aggregates. Rare perivascular CD3^+^ cells were observed in the maternal caruncle, though not associated with pathological changes. Minimal diffuse infiltrate of CD3^+^ cells were also observed in the base of the caruncles of the *N. caninum*-inoculated animals. In 40 to 90% of the placentomes of the *N. caninum*-inoculated animals culled at 28 dpi there was a minimal to mild infiltrate of CD3^+^ cells forming aggregates (in some cases multifocal infiltration) in areas surrounding necrotic foetal villi, necrotic foci and in connective tissue in maternal caruncle. Some of these aggregates were also located in perivascular areas. Rare CD3^+^ cells were also observed in areas of the maternal caruncle where no pathological lesions were present. In 90 to 100% of the collected placentomes from the *N. caninum*-inoculated dams culled at 42 dpi, mild multifocal infiltrate of CD3^+^ cells were located in large areas of necrosis in maternal caruncles, in the caruncular stalk surrounding cryptal epithelium, and in necrotic foetal villi. Some of these infiltrates were also in the perivascular regions of the maternal caruncle. Finally, in 10 and 60% of the placentomes of the *N. caninum*-inoculated dams culled at 56 dpi, mild aggregates of CD3^+^ cells were surrounding necrotic foci (see Figure 1d) in perivascular areas of the maternal caruncles and in necrotic foetal villi. Single rare CD3^+^ cells were observed in the maternal caruncle of negative control animals, not associated with any pathological changes. The mean scores of total T cells in negative control and *N. caninum-*inoculated animals are shown in Figure 3. Statistically significant differences in infiltration score distributions between *N. caninum*-inoculated and negative control animals were found for CD3^+^ cells (*p* = 0.0424).

#### CD4^+^ cells

CD4^+^ cells were only observed in 10% of the placentomes of one out of three *N. caninum*-inoculated dams culled at 14 dpi, forming small aggregates in necrotic foci in the maternal caruncles and surrounding necrotic foetal villi. Some CD4^+^cells were also observed in areas of the maternal caruncle not associated with any pathological features. In the samples taken at 28 dpi, between 10 to 70% of the placentomes from *N. caninum*-inoculated dams had scattered single or small aggregates of CD4^+^ T cells associated with necrotic foci in the maternal caruncles and, in some cases, in the periphery of blood vessels (see Figure 2b). They were also present focally in maternal caruncles, though not associated with pathological changes. In the *N. caninum*-inoculated cows culled at 42 dpi, 40 to 80% of the placentomes had small aggregates of CD4^+^ cells in the maternal caruncle, surrounding necrotic foci and in necrotic foetal villi. Minimal infiltrates of CD4^+^ cells were also observed at the base of caruncles not associated with any pathological changes. In the placentomes of the two *N. caninum*-inoculated animals culled at 56 dpi, a similar pattern of CD4^+^ cell infiltration was observed, but only in 10 to 40% of the sampled tissues, surrounding necrotic areas in maternal caruncles in a perivascular location not associated with lesions. Single CD4^+^ cells were observed in maternal caruncle of negative control animals, with no associated pathological changes. The mean scores of CD4^+^ T cells in negative control and *N. caninum*-inoculated dams are shown in Figure 3. No statistically significant differences in the distribution of scores of CD4^+^ infiltration scores between the *N. caninum*-inoculated and control animals were detected (*p* = 0.1677).

#### CD8^+^ cells

In the three *N. caninum*-inoculated dams culled at 14 dpi, there were rare CD8^+^ T cells surrounding small necrotic foci in maternal caruncles of 10% of placentomes. In all *N. caninum*-inoculated dams at 28 dpi, there were rare to moderate numbers of CD8^+^ T cells around necrotic foci in the maternal caruncle of between 10 and 30% of placentomes (see Figure 2c). In the *N. caninum*-inoculated animals culled on 42 dpi, there were rare CD8^+^ cells near foci of necrosis in the maternal caruncle in 10 to 50% of the placentomes. Rare CD8^+^ cells were associated with small necrotic foci in the maternal caruncle in 10% of the placentomes in one of the two *N. caninum*-inoculated animals culled at 56 dpi. Rare and diffusely distributed CD8^+^ cells were also observed in maternal caruncles of some placentomes of *N. caninum*-inoculated and negative control animals, though not associated with pathological changes. In Figure 3, the mean CD8^+^ T cell scores are summarised. Similarly to CD4^+^ cells, the differences in the distribution of CD8^+^ scores between *N. caninum*-inoculated and negative control groups were not statistically significant (*p* = 0.2548).

#### γδTCR^+^ cells

In 10 to 70% of the selected placentomes from the *N. caninum*-inoculated animals culled at 14 dpi, a minimal to mild γδ-T cell infiltrate was seen in necrotic foci in the maternal caruncles or in necrotic and mineralised foetal villi. In 80 to 90% of the placentomes from three of the *N. caninum*-inoculated animals culled at 28 dpi there was a minimal to mild infiltrate of γδ-T cells in necrotic and mineralised areas of the maternal caruncle, or in necrotic and mineralised foci at the base of the caruncles (see Figure 2d) and in necrotic foetal villi. In 60 to 100% of the placentomes from the *N. caninum*-inoculated animals culled at 42 dpi, there was a minimal to mild focal infiltrate of γδTCR^+^ cells surrounding necrotic and mineralised foci at the base of the caruncles. In the two *N. caninum*-inoculated animals culled at 56 dpi, 70 and 90% of the placentomes, respectively, contained minimal to mild focal infiltrate of γδ-T cells in necrotic foetal villi, in the connective tissue of the foetal-maternal junction and at the base of the maternal caruncles, associated with necrosis. Gamma delta T cells were also diffusely observed at the base of the maternal caruncles, generally not associated with pathological changes in negative control and *N. caninum*-inoculated animals at 14, 28, 42 and 56 dpi. The mean γδ-T cell score for negative control and *N. caninum*-inoculated animals are plotted in Figure 3. Statistically significant differences were found in the distribution of γδ-T cell scores between *N. caninum*-inoculated and negative control animals (*p* = 0.0216).

#### NKp46^+^ cells

Single NKp46^+^ cells were observed throughout the maternal caruncle in the placentomes of all the *N. caninum*-inoculated animals culled at 14, 28, 42 and 56 dpi. In two out of three and in one out of three *N. caninum*-inoculated animals culled at 28 dpi and 42 dpi, respectively, aggregates of NKp46^+^ cells were detected in maternal caruncles surrounding foci of necrosis and mineralisation (see Figure 2e). Minimal infiltrates of NKp46^+^ cells were also observed in all the *N. caninum*-inoculated and negative control animals, though not associated with pathological changes. The NKp46^+^ mean scores are plotted in Figure 3. Statistically significant differences in the distribution of NKp46^+^ infiltration scores were observed between *N. caninum*-inoculated and negative control groups (*p* = 0.0036).

#### CD79_αcy_^+^ cells

Rare individualised CD79_αcy_^+^ cells were observed in all animals from both the *N. caninum*-inoculated and negative control groups on 14, 28, 42 and 56 dpi. These cells were diffusely distributed mainly aligned with the endometrial epithelium in the maternal caruncles and in some foetal villi, but not associated with pathological changes (see Figure 2f). No statistically significant differences in infiltration score distribution were found for CD79_αcy_^+^ cells between the *N. caninum*-inoculated and negative control groups (*p* = 0.8905).

## Discussion

After inoculation with live tachyzoites of the Nc-1 isolate at 210 days of gestation, all dams showed evidence of infection although no abortions occurred and only minimal to mild necrotic and inflammatory lesions were observed in placental and foetal tissues [29]. These findings are similar to previous studies where dams intravenously inoculated with Nc-Liv at the same gestational age gave birth to asymptomatic congenitally infected calves [23,30]. In comparison, the outcome of infection in early gestation is often fatal with extensive lesions [17,23,25,30] while infection in mid-gestation may result in abortion or the birth of persistently infected calves with mild lesions [18,23,25,26,28,35,36].

This paper describes a novel scoring methodology (similar to that described by Tekin and Hansen [32]), in order to allow a more objective analysis and comparison of the immune responses in fixed tissue samples. The protocol could be standardised because we used the same IHC protocols in all the samples and the scoring was undertaken by the same observer. Furthermore, this technique could be modified and applied to other studies with the aim of characterising and compare cellular immune responses in fixed tissue samples.

The results of the phenotypic analysis of the immune cell infiltrate in the placentas of the present study have shown that, although differential infiltration was observed between *N. caninum*-inoculated and negative control animals, no statistically significant differences were found for macrophages. Initially, macrophages were observed in large numbers in the placentas of *N. caninum*-inoculated dams culled at 14 dpi. Although *Neospora* was not detected by PCR or IHC in the placentas of these animals [29] the macrophage infiltration could have been involved in the initiation of an immune response to the parasite challenge in these *N. caninum*-inoculated animals. Monocytes/macrophages are one of the principal cellular components of innate immunity, acting as antigen presenting cells and consequently influencing the functional direction of the subsequent adaptive immune response [37,38]. Indeed Rosbottom et al. [35] demonstrated that endometrial macrophage populations were increased in pregnant cows after experimental infection with *N. caninum*. At 28 dpi, macrophage infiltration was minimal to mild and an increased number of positive cells was observed at 42 and 56 dpi. This infiltration is associated with the presence of more severe pathological changes in the placenta [29]. Macrophages are not only involved in anti-parasitic activity but also play a key role in the tissue repair process, since they are the principal cell type responsible for wound debridement [39]. This may help to explain their presence after the appearance of tissue damage.

Statistical differences between the *N. caninum*-inoculated and the negative control animals were observed for the infiltration of CD3^+^ T cells and, a similar phenomenon was observed for CD4^+^, CD8^+^ and γδ-T cells. A positive correlation was observed between these cellular infiltrates and the presence of pathological changes. The mild inflammatory infiltrates observed in the analysed placentomes of *N. caninum*-inoculated dams primarily expressed CD4^+^ and γδ-T cell markers, indicating a predominant Th1 response. Mean CD8^+^ T cell scores were lower than those for γδ T cells and T helper cells. The time pattern for the appearance of inflammatory cells in the placenta was related to the presence of *Neospora* (PCR and IHC) in the placental samples [29]. Orozco et al. [40] found scattered and fewer CD4^+^ and CD8^+^ T cells in the uterus of pregnant cows naturally infected with *N. caninum* and found no differences when compared with seronegative cattle. However, it has been established that *Neospora* is largely controlled by cell-mediated immune mechanisms and specifically, CD4^+^ T lymphocytes have a significant protective role, demonstrable by the direct lysis of *N. caninum*-infected cells and production of IFN-γ, which can significantly inhibit multiplication of the parasite [41-45]. Following infection with *N. caninum* in cattle, CD4^+^ T lymphocytes are principal components of the Th1 response and produce pro-inflammatory Th1 type cytokines including IFN-γ, TNF-α and IL-12, which have an essential role in protective immunity against the parasite [23,43,45-49]. On the other hand, this Th1 response can be detrimental to pregnancy and can compromise foetal survival [19,38,50]. In vitro studies have shown that treatment of ruminant cells with IFN-γ significantly inhibited intracellular multiplication of *N. caninum*[46].

In humans and ruminants, γδ-T cells are one of the immune cells associated with mucosal surfaces and in the placenta they may be part of the first line of defence against pathogens [20,31,51]. In the peripheral blood and lymphoid organs of young ruminants, they represent up to 50% of all T cells [52], but their role in combating *N. caninum* infections is not yet known [31]. In murine models, γδ-T cells may also have the capacity to trigger foetal losses by reacting against the foetal trophoblast [53]. After *N. caninum* infection in early gestation, placentas contain moderate numbers of γδ-T cells, which can increase to large numbers if dams are carrying dead foetuses [31]. The mild γδ-T cell infiltration that was detected in the present trial supports the hypothesis that, during pregnancy, an anti-*Neospora* maternal immune response at a later stage is less harmful than one that occurs in early gestation.

Natural killer (NK) cells play an important role in the early response to a wide variety of pathogens, including *N. caninum*, and also direct the adaptive immune response towards a Th-1 response. Furthermore, after *N. caninum* infection during early gestation, dams carrying live foetuses show a lower number of NK cells when compared with dams with dead foetuses, suggesting a role for these cells in the immunopathogenesis of neosporosis [31]. During the present trial, mean scores of NK infiltration were relatively constant throughout the period of study, unlike that observed with T cells (CD3^+^, CD4^+^, CD8^+^ and γδ-T cells). The degree of infiltration of NK cells was similar when compared with observations at early gestation in dams carrying live foetuses [31].

In previously reported work, B-cell-deficient mice were shown to be increasingly susceptible to cerebral *N. caninum* infection, suggesting an important role for B cells in host immunity against *N. caninum*[54]. Nevertheless in the present study, only rare or single CD79_αcy_^+^ cells were observed with no differences between negative control and *N. caninum*-inoculated dams, similarly to infection in early gestation [31] and after recrudescence of infection in mid and late-gestation [28]. These data suggest that these cell types are probably not involved in the immunopathogenesis of neosporosis in pregnant cows. However, further analysis will be required in order to elucidate the true identity of the CD79_αcy_^+^ cells, because positive labelled cells morphologically and histologically resembled trophoblast cells instead of B cells. They were located in the trophoblast layer and even though the majority were mononucleated cuboidal cells, occasional binucleated cells were also labelled. More studies using other mAbs need to be carried out in order to establish the presence or absence of B cells in these placentas, and to resolve the identity of these CD79_αcy_^+^ cells.

The observed differences in the pattern of cellular responses at different stages of gestation may be attributable to differences in the immunological environment which allow or hamper the multiplication of the parasite within the placenta. This is further reflected in the clinical outcome of infection at different stages of pregnancy. Previous studies have reported *N. caninum* specific cellular proliferation responses and a corresponding production of IFN-γ early in gestation; while in mid-gestation these immune responses are not as powerful, allowing *Neospora* transmission. This suggests that biological changes associated with pregnancy may allow reactivation of tissue cysts of *N. caninum* leading to the release of bradyzoites [28,55]. The host immune response may also be influenced by hormones produced during pregnancy. Progesterone and prostaglandin E2 are known to bias a T-cell response towards a Th2 phenotype during human pregnancy [56,57]. Steadily increasing levels of progesterone in the plasma of pregnant cattle have been documented from early to mid-gestation; then these values significantly declined in the last few weeks of gestation [58]. Collectively, these observations tend to favour a bias towards a more regulatory Th2-type cytokine *milieu* during normal pregnancy, especially in mid-gestation [59]. In accordance with previous works [19,28], a stronger Th1 response was expected in this study, whereas we observed a milder Th1-type cellular balance compared with responses to infection during early stages of pregnancy [31], possibly resulting in the minor clinical consequences of the disease in late gestation. However, it is important to consider that the immunological maturity of the foetus may also play an important role in influencing the pathogenesis of a *Neospora* infection.

Immunological changes have been reported during normal pregnancy, seemingly playing a critical function in the protection of the foetus and in the “housekeeping” of the placenta [34,60,61]. In the current study no phenotypic differences were observed in the placentas from the negative control animals over a period of 42 days (see Additional file 2) so it seems unlikely that the immune cell infiltrate observed in the placentas of dams inoculated with *Neospora* was simply due to physiological changes of gestation.

No differences were observed for each of the immune cell markers between the negative control animals culled at 28, 42 and 56 dpi when compared with the one negative control animal culled at 14 dpi, which was *N. caninum* positive by PCR [29]. This observation is in agreement with other immunological findings, since this animal was serologically negative and do not respond to *Neospora* antigen in the lymphoproliferation assays (Paul Bartley, unpublished observations).

In conclusion, following infection of pregnant cattle at 210 days of gestation, the immune cell infiltrate in placental tissues was milder than that observed in similar studies that investigated infection at earlier gestational stages and can partially explain the milder clinical outcome, i.e. congenital transmission but no abortions.

Previous reports on neosporosis show an association between production of inflammatory cytokines, such as IFN-γ, TNF-α and IL-12 and disease pathogenesis [28,41-44,46-48,55]. However, it remains unclear which cells are responsible for the production of these cytokines and how the cells and cytokines relate to pathogenesis, particularly at different stages of gestation. Further studies are needed in order to clarify this aspect of the disease.

## Competing interests

The authors declare that they have no competing interests.

## Authors’ contributions

FC, FK and EAI conceived this study and participated in its design and coordination. JB, FK, SWM, PMB, YP, MR, FC and EAI participated in the necropsy and sampling of the animals. JPA performed the statistical analysis. GC carried out the IHC analysis of the samples and has written the manuscript; with inputs form all authors. All authors read and approved the final manuscript.

## Supplementary Material

Additional file 1 **mAb used to characterize the different immune cell populations in the placentomes from the experiment.** Table showing details of each of the mAb used to label monocytes/macrophages, T cells, NK cells and B cells [62-70]. Click here for file

Additional file 2 **Mean of the infiltration scores of the different phenotype of inflammatory cells on placentomes.** CD68^+^ (macrophages), CD3^+^ (total T cells), CD4^+^ (T helper), CD8^+^ (cytotoxic T cells), γδTCR^+^ (γδ T cells), NKp46^+^ (NK cells) and CD79_αcy_^+^ labelled cells in the placentas. Numbers in the horizontal axis represent days post inoculation (dpi). Error bars for the inoculated animals indicate standard error of the means (SEM) (for negative control animals no SEM could be generated because there were only single animals at each time point). Click here for file
