# Supplementary material for: Phenotypic characterisation of the cellular immune infiltrate in placentas of cattle following experimental inoculation with Neospora caninum in late gestation
Source: Vet Res. 2013 Jul 22;44(1):60. doi: 10.1186/1297-9716-44-60 (PMC3726360; doi:10.1186/1297-9716-44-60)
Supplement: Additional file 1 — mAb used to characterize the different immune cell populations in the placentomes from the experiment. Table showing details of each of the mAb used to label monocytes/macrophages, T cells, NK cells and B cells [62-70]. [file 1297-9716-44-60-S1.docx]

| **Cluster of differentiation** | **mAb clone** | **Targeted immune cell** | **Dilution** | **References** |
| --- | --- | --- | --- | --- |
| CD68 | EBM11^(1)^ | Monocytes/macrophages | 1:100 | [32,34,40,63] |
| CD3 | MM1A^(2)^ | Total T cells | 1:2000 | [31,60,62,66] |
| CD4 | CC30^(3)^ | T helper cells | 1:50 | [63-65] |
| CD8 | CC58^(3)^ | Cytotoxic T cells | 1:200 | [65-67] |
| γδTCR | IL-A29^(2)^ | γδ-T cells | 1:4000 | [31,62,65,68] |
| CD335 | NKp46^(3)^ | Natural killer cells | 1:250 | [31,69] |
| CD79_αcy_ | HM57^(1)^ | Total B cells | 1:100 | [31,70] |

1. Dako Cytomation, Glostrup, Denmark
2. VMRD Inc, Washington, USA
3. AbD Serotec, Oxford, UK
