# Supplementary figures and images for: Phenotypic characterisation of the cellular immune infiltrate in placentas of cattle following experimental inoculation with Neospora caninum in late gestation
Source: Vet Res. 2013 Jul 22;44(1):60. doi: 10.1186/1297-9716-44-60 (PMC3726360; doi:10.1186/1297-9716-44-60)

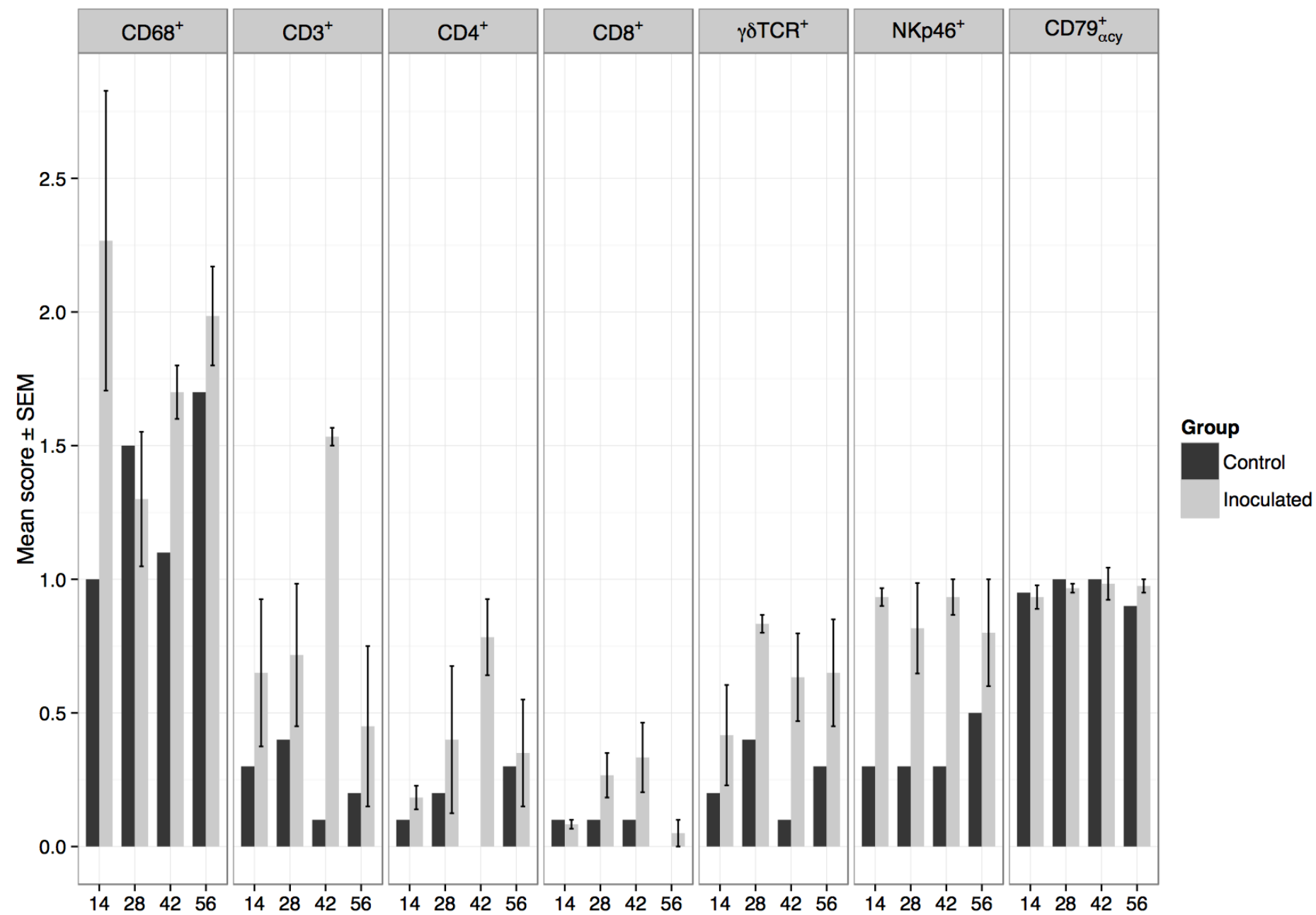

Supplement: Additional file 2 — Mean of the infiltration scores of the different phenotype of inflammatory cells on placentomes. CD68+ (macrophages), CD3+ (total T cells), CD4+ (T helper), CD8+ (cytotoxic T cells), γδTCR+ (γδ T cells), NKp46+ (NK cells) and CD79αcy+ labelled cells in the placentas. Numbers in the horizontal axis represent days post inoculation (dpi). Error bars for the inoculated animals indicate standard error of the means (SEM) (for negative control animals no SEM could be generated because there were only single animals at each time point). [file 1297-9716-44-60-S2.pdf]
